# Laser-Induced Periodic Nanostructure on Polyimide Film Surface Using 248 nm Excimer Laser

**DOI:** 10.3390/nano15100742

**Published:** 2025-05-15

**Authors:** Songqing Zhao, Xuan Xie, Mingyang Li, Limin Yang, Tongjing Liu

**Affiliations:** 1 School of Science and Arts, China University of Petroleum-Beijing at Karamay, Karamay 834000, Chinalimingyang7278@gmail.com (M.L.); ylm@cup.edu.cn (L.Y.); 2 College of Science, China University of Petroleum, Beijing 102249, China; 3 School of Petroleum, China University of Petroleum-Beijing at Karamay, Karamay 834000, China

**Keywords:** laser-induced periodic surface structure, polyimide, nanostructure, excimer laser, wettability

## Abstract

In this study, nanoscale periodic surface structures were fabricated on polyimide (PI) films using a linearly polarized KrF excimer laser with a wavelength of 248 nm. The effects of laser energy density and pulse number on the morphology and surface roughness of laser-induced periodic surface structures (LIPSSs) were systematically investigated. When the pulse width was 20 ns, the repetition rate was 10 Hz, and the beam incidence angle was normal (90°), periodic ripples with a spatial period of approximately 200 nm formed within an energy density range of 7–18 mJ/cm^2^ and pulse number range of 6000–18,000. The most uniform and well-defined structures were achieved at 14.01 mJ/cm^2^ and 12,000 pulses, with a ripple depth of 60 nm and surface roughness ($R_{a}$) approximately 26 times greater than that of pristine PI. The ripple orientation was consistently perpendicular to the laser polarization, consistent with low-spatial-frequency LIPSS (LSFL) formation mechanisms governed by interference-induced photothermal effects. In addition, surface wettability was found to be significantly enhanced due to changes in both surface chemistry and topography, with the water contact angle decreasing from 73.7° to 19.7°. These results demonstrate the potential of UV nanosecond laser processing for the scalable fabrication of functional nanostructures on polymer surfaces for applications in surface engineering and biointerfaces.

## 1. Introduction

Laser processing boasts advantages such as cleanliness, speed, selectivity, and non-contact operation. During laser-based surface modification, a unique class of periodic structures—laser-induced periodic surface structures (LIPSSs)—have been widely observed and are now regarded as universal phenomena [1]. LIPSSs can form on nearly all types of solid surfaces when the energy accumulated from laser irradiation surpasses the ablation threshold of the target material [2]. In recent years, LIPSSs have been extensively reported on metals [3,4,5,6,7,8,9] and oxides [10,11,12,13], with growing interest in their functionalization for practical applications.

Notably, LIPSSs on metallic substrates have enabled advances in biomedical engineering, wetting control, and optical functionality. For example, Gupta et al. [14] demonstrated that micro/nano-hierarchical LIPSS patterns on Ti6Al4V significantly enhanced fibroblast cell adhesion (230% improvement) and antibacterial efficacy (81.5% bacterial death rate), highlighting their potential for implantable devices. Similarly, Wang et al. [15] investigated wettability tuning using LIPSSs on various metal interfaces, showing enhanced interfacial control for Sn-based alloys. Moreover, laser structuring has been extended to semiconductors, as shown by Li et al. [16], who used femtosecond laser scanning to inscribe structural color patterns on monocrystalline silicon with polarization-controlled LIPSSs, providing a platform for anti-counterfeiting and optical data storage.

In contrast, the exploration of LIPSSs on polymeric surfaces remains comparatively underdeveloped [17,18,19,20,21,22,23,24]. Among these materials, polyimide (PI) films are particularly attractive due to their excellent thermal and chemical stability, low thermal conductivity, high mechanical strength, and flame retardancy, which render them suitable for precise and localized laser modification [25]. Unlike metals, PI requires only that the surface temperature briefly exceeds its glass transition temperature ($T_{g}$) to enable LIPSS formation, making it highly responsive to laser-induced structuring.

Despite these advantages, LIPSSs formed on polymeric substrates often suffer from low uniformity, poor reproducibility, and difficulty in achieving large-area periodicity. The underlying formation mechanisms remain a topic of debate, involving competing theories, including surface plasmon polariton excitation, interference of incident and scattered waves, and thermocapillary instabilities [26]. Furthermore, polymer-specific factors such as thermal diffusivity, photothermal response, and laser absorption behavior contribute to significant complexity [27,28,29].

Although LIPSSs have demonstrated potential in fabricating functional surfaces, many previous studies have lacked systematic investigations into the effects of laser parameters—particularly pulse number, fluence, scanning path, and beam overlap—on structure evolution in PI. For instance, Sun et al. [30] achieved nanogroove formation on PI via femtosecond laser, without evaluating parameter robustness. Li et al. [31] investigated post-thermal treatment to refine structure regularity, but did not expand to varied fluences or pulse regimes. Moreover, studies focusing on long-range periodicity, multi-scale structuring, or process scalability remain rare [32].

In this context, the interplay between DLIP (Direct Laser Interference Patterning) and LIPSSs offers new opportunities for controllable structuring. However, such techniques have been primarily validated on metals and oxides, with limited application to polymers like PI. A theoretical understanding of transitions between LSFL and HSFL regimes, and their dependence on polarization, angle of incidence, and multi-pulse accumulation, also remains incomplete for polymer systems. These limitations highlight the need for further research to improve control, scalability, and structural regularity in PI-based LIPSS fabrication.

In precisely tuning laser parameters and optical paths, high-resolution periodic nanostructures can nonetheless be fabricated on PI and other polymer surfaces. Localized modification is also achievable via mechanical stage control or mask-assisted exposure. These capabilities make LIPSS a highly attractive technique for polymer surface functionalization, with promising applications in cell alignment and tissue engineering [33,34,35,36], surface-enhanced Raman scattering (SERS) [37], and organic electronics [38,39,40].

To fully exploit these advantages and improve the controllability and uniformity of LIPSSs on polyimide surfaces, a more detailed investigation into the relationship between laser parameters and surface morphology is required. Specifically, the generation of LIPSSs on polymers depends on the localized surface temperature exceeding the material’s glass transition temperature, and the process is highly sensitive to parameters such as laser fluence, pulse number, and angle of incidence. In this study, we employed a 248 nm KrF nanosecond excimer laser to fabricate nanoscale periodic ripple structures on PI films. The effects of pulse number and energy density on the resulting surface morphology were systematically investigated using atomic force microscopy (AFM), and the optimal laser conditions for achieving uniform and well-defined LIPSSs were identified.

## 2. Experiment

### 2.1. Optical Path Design and Sample Preparation

**Figure 1:**
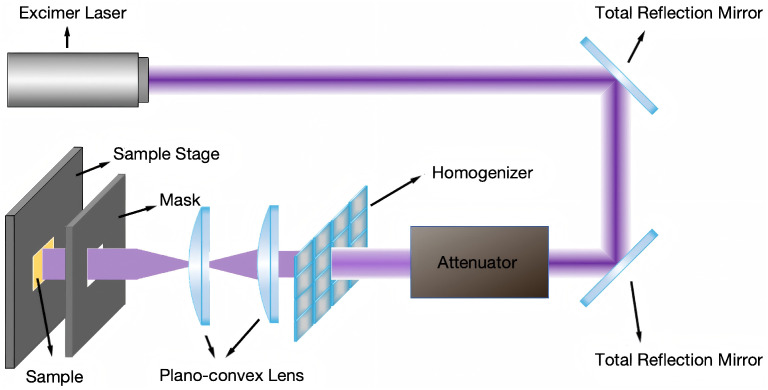
Schematic of the optical path system for the excimer laser.

As shown in Figure 1, an optical path system matching the excimer laser was designed to achieve a more uniform distribution of laser pulse energy and to regulate the energy magnitude of laser pulses. Two total reflection mirrors were used to change the direction of the optical path, conserving horizontal space. An energy attenuator was then placed to decrease the energy of the original laser pulses and adjust the energy magnitude of the laser pulses by altering the angle of the attenuating mirror inside. A homogenizer consisting of a beam splitter and two plano-convex lenses was positioned behind the attenuator. Its function is to transform Gaussian-distributed laser pulses into high-flat distributions, making the energy distribution of the laser pulses more uniform. Additionally, in swapping plano-convex lenses with different focal lengths, the spot size of the laser pulses can be adjusted. Finally, a mask and sample stage were placed at the focus of the second plano-convex lens. The KrF excimer laser emits laser pulses with an energy density of 0–50 mJ/cm^2^ based on this optical path system. The 248 nm KrF excimer laser used in this study emits inherently linearly polarized light, a characteristic of its transverse electric (TE) mode operation. The polarization direction is fixed perpendicular to the long axis of the rectangular beam profile and remained unchanged during all experiments. Since the native beam polarization was stable and well defined, no additional polarizing optics were required. This fixed polarization direction is particularly relevant to the formation of LIPSSs, as the ripple structures were consistently observed to orient perpendicular to the laser polarization, in agreement with the typical behavior of low-spatial-frequency LIPSSs (LSFLs). Other laser parameters can be altered through the laser controller. The laser fluence values used in this study, such as 14.01 mJ/cm^2^, were not arbitrarily selected. Instead, they were determined by precise measurements using a calibrated laser energy meter positioned after the attenuator in the optical path. The attenuator allows for continuous adjustment of output energy via a rotatable reflective plate, enabling fine control over the energy density incident on the sample. As a result, the actual fluence values are not always round numbers but reflect the true, measurable output under each condition. Among the tested settings, 14.01 mJ/cm^2^ consistently yielded the most regular and well-defined periodic surface structures. This value was repeatedly verified and used as a stable reference point in our parameter optimization. Although not a nominal round number, it represents a reproducible and experimentally significant setting within the effective fluence range for polyimide surface structuring.

This experiment used a polyimide (PI) film with a density of 1.45 g/cm^3^, a glass transition temperature $T_{g}={385\;}^{\circ }C$, and a thickness of 0.05 mm. Before laser irradiation, the PI film was cut into square slices with dimensions of 15 mm × 15 mm. The samples were then immersed in absolute ethanol and deionized water, respectively, and ultrasonically cleaned for 10 min. Finally, they were dried in a vacuum drying box. The PI film was fixed on the sample stage and irradiated with laser pulses (λ=248nm, $t_{p}=20\;\mathrm{ns}$, f=10Hz) to prepare different PI samples by adjusting the pulse number and laser energy density.

### 2.2. Sample Characterization

A Cypher VRS-type atomic force microscope (AFM, Oxford Instruments) was used to observe the surface morphology of the samples. The tapping mode was employed to ensure stable, high-resolution imaging with minimal sample damage. The surface roughness (Ra) was calculated as the arithmetic average of the absolute deviations from the mean line within the scanned area, following ISO 21920-2 [41]. For each sample, three separate 5 μm × 5 μm areas were scanned, and the average Ra value was reported to ensure consistency and statistical reliability.

## 3. Results and Discussion

### 3.1. Effect of Pulse Number on LIPSS-PI Surface Morphology

**Figure 2:**
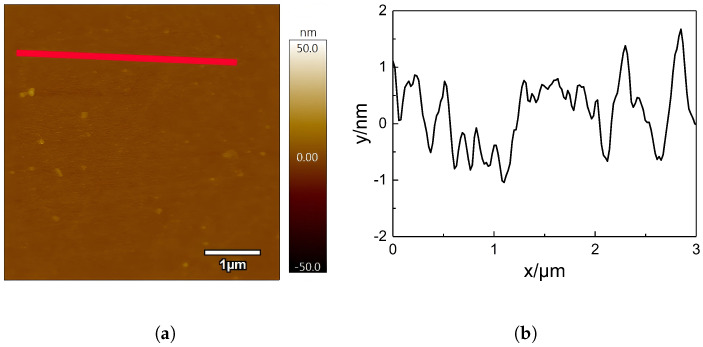
AFM image and corresponding height profile. (**a**) AFM image of untreated PI. (**b**) Height variation in the red line in (**a**).

Figure 2a is an AFM image of the untreated PI film, with Ra representing the surface roughness of 0.550 nm. With reference to the scale on the right, it can be found that the untreated PI surface is not completely flat and smooth, with many small protrusions. This is due to the reaction agent added during the synthesis of PI not reacting completely and remaining in the polyimide. Figure 2b shows a height profile of the red line in Figure 2a. The height change range of the untreated PI film surface is about 3 nm, which is consistent with the information reflected in Figure 2a.

**Figure 3:**
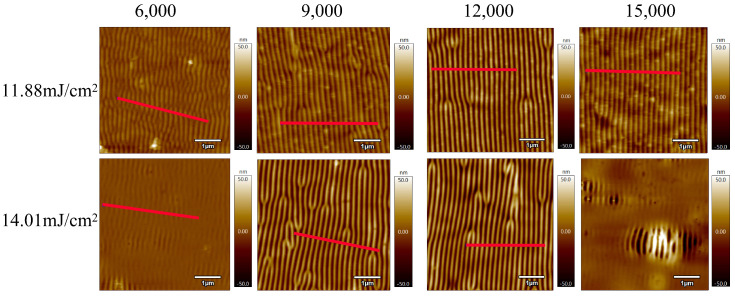
AFM images of laser-treated PI thin films with energy densities of 11.88 mJ/cm^2^ and 14.01 mJ/cm^2^ after treatment with different pulse numbers. The laser was linearly polarized, and the resulting LIPSSs are oriented perpendicular to the polarization direction, consistent with the typical behavior of low-spatial-frequency LIPSSs (LSFLs).

In order to study the effect of pulse number on the morphology of laser-induced periodic surface structures (LIPSSs) on polyimide (PI) films, samples were irradiated at energy densities of 11.88 mJ/cm^2^ and 14.01 mJ/cm^2^ with pulse numbers of 6000, 9000, 12,000, and 15,000, respectively. Figure 3 shows their corresponding AFM images. Under these conditions, all treated PI film samples exhibited periodic surface structures. Comparison reveals that as the pulse number increased from 6000 to 12,000, the regularity of the ripple structures improved. However, a further increase to 15,000 pulses led to structural deterioration. When the energy density was 14.01 mJ/cm^2^ and the pulse number reached 15,000, only local periodic structures remained visible on the PI surface. As previously discussed, the formation of LIPSSs on polymer surfaces requires the accumulated energy to surpass the ablation threshold of the material. This observation suggests that the cumulative energy under these conditions significantly exceeded the ablation threshold of PI, resulting in damage to the regular structure. The presence of isolated ripple regions indicates that despite improvements to the optical path system, slight non-uniformities in energy distribution still existed across the irradiated area. Furthermore, on PI surfaces with striped patterns, some regions displayed tree-like bifurcations, where one stripe splits into two or two merge into one. These features were consistently located at surface protrusions. Through comparisons with Figure 2, it can be inferred that these protrusions locally disrupt the energy distribution, leading to the formation of irregular tree-like LIPSS features.

To better understand the formation mechanism of LIPSSs on the PI surface, it is important to consider the material’s laser ablation threshold. Although we did not directly measure the single-pulse ablation threshold in this study, prior research reports that for polyimide irradiated with a 248 nm nanosecond laser, the ablation threshold typically lies between 10 and 12 mJ/cm^2^. Our experimental results are consistent with this range. Moreover, it is known that the effective ablation threshold decreases with an increasing number of successive laser pulses, due to cumulative thermal effects, incubation, and local bond weakening. This is reflected in our observations: even at energy densities slightly below the reported single-pulse threshold, ripple structures were formed after sufficient pulse accumulation (6000–12,000 pulses), indicating that the material’s surface undergoes gradual modifications that lower the energy required for subsequent ablation. This cumulative effect plays a critical role in enabling LIPSS formation under conditions that would otherwise be sub-threshold for single-pulse ablation.

It is worth noting that the orientation of the laser-induced periodic surface structures (LIPSSs) is consistently perpendicular to the laser polarization direction. This observation agrees with the expected characteristics of low-spatial-frequency LIPSSs (LSFLs), which typically form orthogonally to the linear polarization of the incident beam.

**Figure 4:**
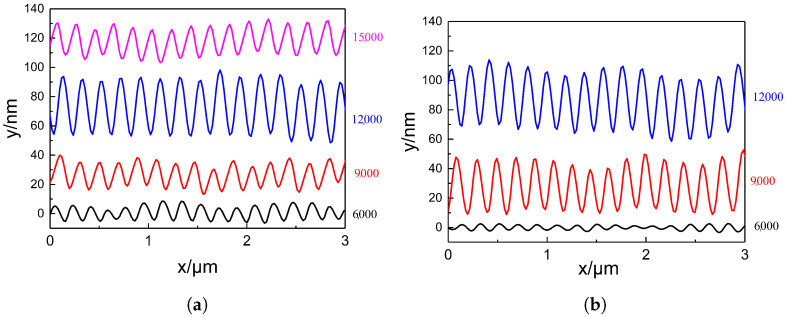
Surface height profiles of PI film treated with different pulse numbers under two energy densities. (**a**) Surface height profiles at 11.88 mJ/cm^2^. (**b**) Surface height profiles at 14.01 mJ/cm^2^.

The two graphs in Figure 4 show the surface height profiles along the red line regions indicated in Figure 3, under two different energy densities. These profiles reveal the variation in surface height of the PI films after laser treatment. As the pulse number increases from 6000 to 12,000, the ripple structures on the PI surface become progressively deeper. However, when the pulse number is further increased to 15,000, the ripple depth decreases. At an energy density of 14.01 mJ/cm^2^ and a pulse number of 12,000, the ripple depth reaches a maximum of approximately 50 nm. Regardless of whether the energy density is 11.88 mJ/cm^2^ or 14.01 mJ/cm^2^, the number of ripple peaks observed within a scan length of 3 μm is consistently 15 or 16. This suggests that the pulse number has minimal influence on the spatial period of the ripple structures. The calculated period lies within the range of approximately 188–200 nm.

**Figure 5:**
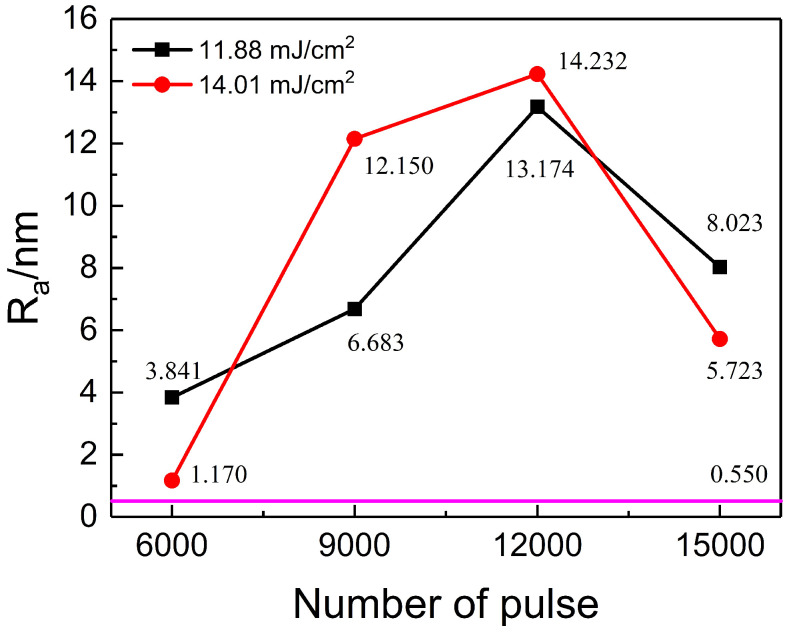
The changes in surface roughness of PI films treated with different pulse numbers at energy densities of 11.88 mJ/cm^2^ and 14.01 mJ/cm^2^.

The surface roughness changes in PI films treated at different pulse numbers and energy densities (11.88 mJ/cm^2^ and 14.01 mJ/cm^2^) are shown in Figure 5. The purple horizontal line represents the surface roughness of the untreated PI film, which is 0.550 nm. At an energy density of 11.88 mJ/cm^2^, the surface roughness increases with the pulse number from 3.841 nm to a peak of 13.174 nm (at 12,000 pulses), and then decreases to 8.023 nm at 15,000 pulses. A similar trend is observed at 14.01 mJ/cm^2^, where the roughness also peaks at 12,000 pulses. Overall, laser irradiation significantly increases the surface roughness of PI films. The maximum roughness of 14.232 nm, obtained at 14.01 mJ/cm^2^ and 12,000 pulses, is approximately 26 times greater than that of the untreated film.

The formation of the ripple structures observed on the PI surface can be attributed to the classical mechanism of low-spatial-frequency LIPSSs (LSFLs). The measured periodicity of approximately 200 nm and the orientation perpendicular to the laser polarization indicate that these structures result from the interference between the incident laser beam and light scattered by surface inhomogeneities. This interference creates a modulated spatial energy distribution, leading to localized heating and periodic material modification.

In polyimide, which does not require melting for surface restructuring, the LIPSS formation is primarily governed by photothermal effects. When the local surface temperature exceeds the glass transition temperature ($T_{g}$), polymer chain mobility increases temporarily, enabling thermally driven topographical deformation. With successive laser pulses, cumulative energy absorption and incubation effects enhance this modulation process. As a result, the observed periodic morphology and the significant increase in surface roughness are manifestations of this self-organized photothermal feedback mechanism.

### 3.2. Influence of Energy Density on LIPSS-PI Surface Morphology

**Figure 6:**
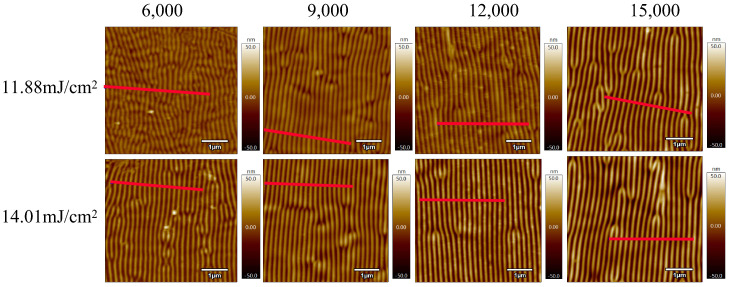
AFM images of PI film treated with different energy densities under pulse numbers of 9000 and 12,000.

Based on the observed influence of pulse number on the surface morphology of LIPSS-PI, pulse numbers of 9000 and 12,000 were selected as optimal parameters for further analysis of the effect of laser energy density. Energy densities of 7.71, 9.86, 11.88, and 14.01 mJ/cm^2^ were investigated. Figure 6 presents AFM images of the PI film surface treated under these conditions. As the energy density increases from 7.71 to 14.01 mJ/cm^2^, the surface ripples become progressively more distinct and isolated from the initial curved background features. Among all samples, the ripple structures generated at 14.01 mJ/cm^2^ with 12,000 pulses exhibit the highest degree of regularity and definition. To further clarify the rationale behind the selected fluence range used in this study, the following observations are noted. Although the laser system used in this study was capable of delivering energy densities up to 50 mJ/cm^2^, the experimental results show that only a narrower fluence window between approximately 7 and 18 mJ/cm^2^ was suitable for generating well-defined periodic surface structures on PI films. Below 7 mJ/cm^2^, no surface modification or ripple formation was observed, indicating that the energy was insufficient to exceed the ablation threshold or trigger the necessary photothermal effects. In contrast, when the energy density exceeded 18 mJ/cm^2^, the PI surface exhibited signs of thermal damage, such as irregular ablation, melting, and structural breakdown, with a complete loss of periodicity. Therefore, in this study, we focus our analysis on the optimal fluence range of 7.71–14.01 mJ/cm^2^, which consistently produced regular and repeatable LIPSSs with controllable morphology. This range was selected to ensure comparability and to avoid artifacts introduced by surface overprocessing or underexposure.

**Figure 7:**
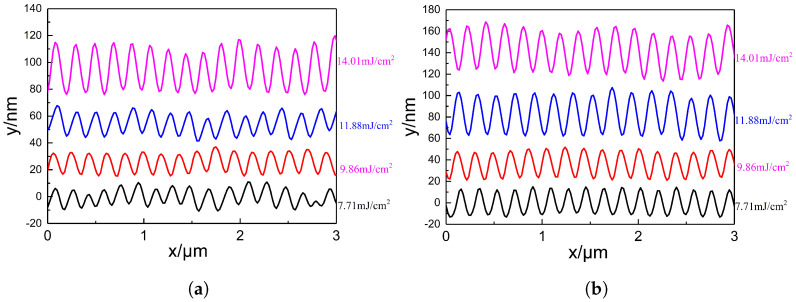
Surface height profiles of PI film treated with different energy densities under two pulse numbers. (**a**) Surface height profiles under 9000 pulses. (**b**) Surface height profiles under 12,000 pulses.

The two graphs in Figure 7 show the surface height profiles of the red line regions in Figure 6 under two different pulse numbers, providing information on the height changes of the PI film surface. Under both pulse numbers, as the energy density increases, the ripple depth on the PI film surface also increases. This indicates that before the accumulated energy reaches the material’s threshold, energy density and pulse number have similar effects on the laser-induced surface periodic structure, both deepening the formed ripple depth. As can be seen from Figure 7, regardless of whether the pulse number is 9000 or 12,000, the number of peaks in the generated ripple structure within a length range of 3 μm is either 15 or 16. This suggests that energy density also has little effect on the period of the ripple structure, which is similar to the effect of the pulse number on the period of the structure.

**Figure 8:**
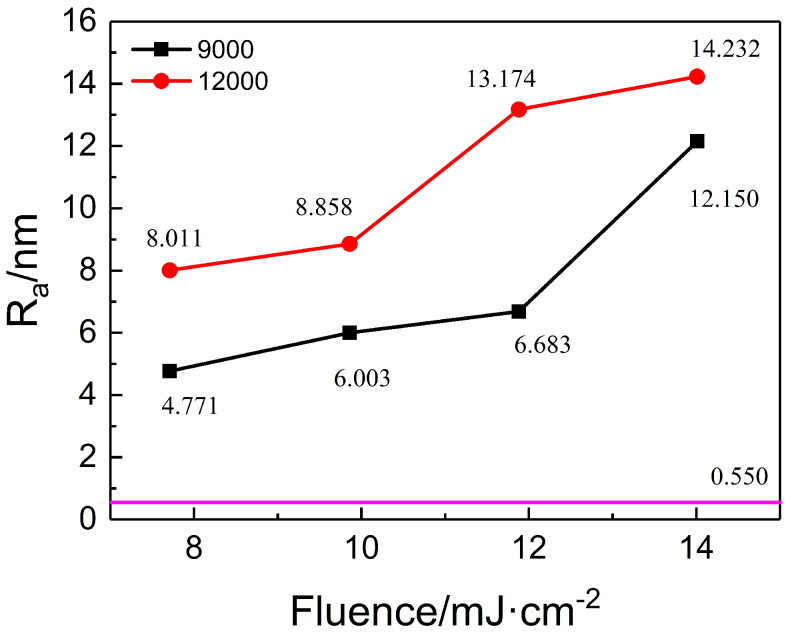
Surface roughness changes in PI film treated with different energy densities under pulse numbers of 9000 and 12,000.

The changes in surface roughness of PI films treated at different energy densities, with pulse numbers of 9000 and 12,000, are shown in Figure 8. At a pulse number of 9000, the surface roughness increases from 4.771 nm to 12.150 nm as the energy density rises from 7.71 to 14.01 mJ/cm^2^. Similarly, at a pulse number of 12,000, the roughness increases from 8.011 nm to a maximum of 14.232 nm across the same fluence range. By comparing Figure 4, Figure 5 and Figure 7, it is evident that the trends in surface roughness correlate closely with changes in ripple depth. In general, deeper ripple structures correspond to higher surface roughness, indicating that both parameters are strongly influenced by laser energy input.

### 3.3. Functional Implications of LIPSS-Modified PI Films for Wettability Control

**Figure 9:**
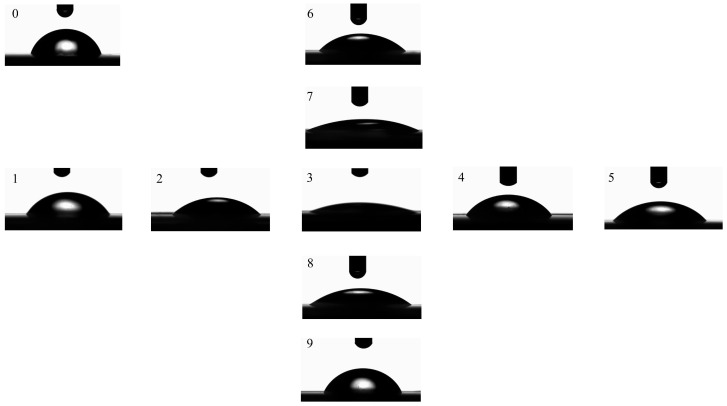
Contact angles of different PI film surfaces under various laser treatment conditions. Numbers “0–9” correspond to samples irradiated at different fluence and pulse numbers, “1–5” represent samples treated with increasing pulse numbers at a fixed fluence of 14.01 mJ/cm^2^, while “6–9” represent samples irradiated with varying fluence levels at a fixed pulse number of 12,000.

To evaluate the functional influence of the laser-induced periodic surface structures (LIPSSs), we investigated changes in the surface wettability and chemistry of the modified PI films (Figure 9).

**Figure 10:**
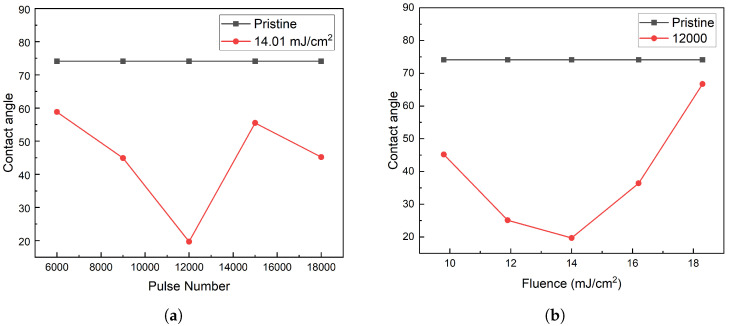
Contact angle results under different laser treatment parameters. (**a**) Contact angles under fixed fluence (14.01 mJ/cm^2^) with varying pulse numbers. (**b**) Contact angles under fixed pulse number (12,000) with varying fluences.

Figure 10 shows the measured water contact angles under different laser processing conditions. The untreated PI film exhibited a contact angle of 73.7°, indicating mild hydrophilicity. After laser modification, the contact angle significantly decreased in all samples. The lowest angle was measured as 19.7° in the sample treated with a pulse number of 12,000 and a fluence of 14.01 mJ/cm^2^—corresponding to the condition where ripple structures were the most regular and deepest.

The observed changes in wettability are attributed not only to surface morphology but also to the modified surface chemistry. Energy-dispersive X-ray spectroscopy (EDS) analysis (Table 1) indicates an increase in oxygen and nitrogen content after laser treatment, likely due to the formation of C–O and C–N groups. These polar functionalities are known to enhance hydrophilicity.

These results suggest that laser-induced periodic nanostructures offer an effective and reproducible route for tailoring the surface properties of PI films. The ability to enhance hydrophilicity in a controlled manner is of great value for applications such as biointerface engineering (e.g., cell adhesion regulation), microfluidic surface modification, and sensor substrates.

## 4. Conclusions

In this work, we employed a KrF excimer laser (248 nm) with a custom-designed optical path system to systematically investigate the effects of pulse number and energy density on the formation of laser-induced periodic surface structures (LIPSSs) on polyimide (PI) films. Under a laser frequency of 10 Hz and normal incidence (90°), ripple-like periodic micro/nanostructures with a spatial period of approximately 200 nm were successfully fabricated within an energy density range of 7–14 mJ/cm^2^ and pulse number range of 6000–15,000.

The results demonstrate that both pulse number and fluence strongly affect the morphology and surface roughness of the PI films. The ripple depth and roughness initially increase with increasing energy input, reaching a maximum at 12,000 pulses and 14.01 mJ/cm^2^. This behavior is attributed to cumulative photothermal effects, where repeated irradiation raises the local temperature above the glass transition threshold, enhancing polymer chain mobility and enabling periodic material rearrangement. Beyond this optimal condition, excessive energy leads to thermal degradation, surface melting, and ripple collapse, resulting in a decrease in structural definition. The formation of LIPSSs is primarily governed by the interference between the incident laser beam and surface-scattered waves, producing a periodic energy modulation that drives self-organized structuring. In polyimide, the mechanism is further dominated by photothermal softening rather than melting, making it highly sensitive to laser parameters. The orientation of the ripples remains perpendicular to the laser polarization, confirming their classification as low-spatial-frequency LIPSSs (LSFLs). Additionally, tree-like bifurcated ripple patterns were observed near surface protrusions. These features are likely caused by local field enhancement at height irregularities, which distorts the interference field and leads to bifurcated energy deposition patterns.

This study establishes a reproducible and scalable method for generating well-ordered LIPSSs on polymer substrates using ultraviolet nanosecond lasers. The results offer valuable insights for the surface engineering of polymeric materials with potential applications in flexible electronics, wetting control, and biointerface design.

## Figures and Tables

**Table 1:** Chemical composition comparison of untreated PI and laser-structured sample (sample 4-3).

Sample	C (at%)	N (at%)	O (at%)	O/C (%)	N/C (%)
Pristine PI	59.89	8.13	31.97	53.38	13.57
Sample 4-3	54.83	12.43	32.74	59.71	22.67

## Data Availability

Data will be made available on request.

## References

[1] Bonse J., Höhm S., Kirner S.V., Rosenfeld A., Krüger J. (2017). Laser-induced periodic surface structures—A scientific evergreen. IEEE J. Sel. Top. Quantum Electron..

[2] Zhang W., Cheng G.H., Feng Q., Cao L., Wang F.P., Hui R.Q. (2011). Abrupt transition from wavelength structure to subwavelength structure in a single-crystal superalloy induced by femtosecond laser. Appl. Surf. Sci..

[3] Liu K.J., Li X.H., Xie C.X., Wang K., Zhou Q., Qiu R. (2017). Formation of sub-200 nm nanostructure on Fe film irradiated by femtosecond laser. Opt. Laser Technol..

[4] Maragkaki S., Derrien T.J.Y., Levy Y., Bulgakova N.M., Ostendorf A., Gurevich E.L. (2017). Wavelength dependence of picosecond laser-induced periodic surface structures on copper. Appl. Surf. Sci..

[5] Lim H.U., Kang J., Guo C.L., Hwang T.Y. (2018). Manipulation of multiple periodic surface structures on metals induced by femtosecond lasers. Appl. Surf. Sci..

[6] Lin X.M., Li X.H., Zhang Y.B., Xie C.X., Liu K.J., Zhou Q. (2019). Periodic structures on germanium induced by high repetition rate femtosecond laser. Opt. Laser Technol..

[7] Giannuzzi G., Gaudiuso C., Di Franco C., Scamarcio G., Lugarà P.M., Ancona A. (2019). Large area laser-induced periodic surface structures on steel by bursts of femtosecond pulses with picosecond delays. Opt. Laser Eng..

[8] Jalil S.A., Yang J.J., Elkabbash M., Singh S.C. (2019). Maskless formation of uniform subwavelength periodic surface structures by double temporally-delayed femtosecond laser beams. Appl. Surf. Sci..

[9] Lee K., Ki H. (2019). Femtosecond laser patterning based on the control of surface reflectance. Appl. Surf. Sci..

[10] He R., Ma H.L., Zheng J.H., Han Y.M., Lu Y.F., Cai C.B. (2016). Periodic structure with a periodicity of 2–3.5 μm on crystalline TiO_2_ induced by unpolarized KrF excimer lasers. Appl. Phys. A.

[11] Nurnberger P., Reinhardt H.M., Kim H.C., Pfeifer E., Kroll M., Müller S., Yang F., Hampp N. (2017). Orthogonally superimposed laser-induced periodic surface structures (LIPSS) upon nanosecond laser pulse irradiation of SiO_2_/Si layered systems. Appl. Surf. Sci..

[12] Ehrhardt M., Lorenz P., Han B., Zhu R., Zimmer K. (2018). Laser-Induced Backside Wet Etching of SiO_2_ with a Visible Ultrashort Laser Pulse by Using KMnO_4_ Solution as an Absorber Liquid. J. Laser Micro Nanoen..

[13] Reinhardt H.M., Maier P., Kim H.C., Rhinow D., Hampp N. (2019). Nanostructured Transparent Conductive Electrodes for Applications in Harsh Environments Fabricated via Nanosecond Laser-Induced Periodic Surface Structures (LIPSS) in Indium-Tin Oxide Films on Glass. Adv. Mater. Interfaces.

[14] Gupta R., Gaddam A., Hema N.A., Prajapati D., Dimov S., Bhatia D., Mishra A., Sofronov Y., Vadali M. (2025). Improving the cell adhesion and antibacterial behaviour on Ti6Al4V through micro and nano hierarchical laser surface texturing. Surf. Innov..

[15] Wang Y., Liu W., Xiao B., Liang X., Lv P., Zhou J., Lin F. (2025). Wettability of Sn alloys at metal interfaces: Metal surface treatment, interfacial temperature control and elemental modification. Surf. Coat. Technol..

[16] Li Z., Chen H., Han M., Yang X., Bai S. (2025). Femtosecond Laser-Induced Grid-like Periodic Surface Structure on Silicon Substrate and Its Preliminary Application. Chin. J. Lasers.

[17] Mezera M., Bonse J., Römer G.-W.R.B.E. (2019). Influence of Bulk Temperature on Laser-Induced Periodic Surface Structures on Polycarbonate. Polymers.

[18] Rebollar E., Hernández M., Sanz M., Perez S., Tiberio A. (2015). Laser-induced surface structures on gold-coated polymers: Influence of morphology on surface-enhanced Raman scattering enhancement. J. Appl. Polym. Sci..

[19] Stofik M., Semeradova A., Maly J., Kolska Z., Nedela O., Wrobel D., Slepicka P. (2015). Direct immobilization of biotin on the micro-patterned PEN foil treated by excimer laser. Colloid Surf. B.

[20] Cui J., Rodriguez-Rodriguez A., Hernández M., García-Gutierrez M.C., Nogales A., Castillejo M., Gonzalez D.M., Muller-Buschbaum P., Ezquerra T.A., Rebollar E. (2016). Laser-Induced Periodic Surface Structures on P3HT and on Its Photovoltaic Blend with PC_71_BM. ACS Appl. Mater. Interfaces.

[21] Michaljanicova I., Slepicka P., Rimpelova S., Kasalkova N.S., Svorcik V. (2016). Regular pattern formation on surface of aromatic polymers and its cytocompatibility. Appl. Surf. Sci..

[22] Nedela O., Slepicka P., Sajdl P., Vesely M., Svorcik V. (2017). Surface analysis of ripple pattern on PS and PEN induced with ring-shaped mask due to KrF laser treatment. Surf. Interface Anal..

[23] Slepicka P., Nedela O., Kasalkova N.S., Sajdl P., Svorcik V. (2017). Periodic nanostructure induced on PEN surface by KrF laser irradiation. Int. J. Nanotechnol..

[24] Nedela O., Slepicka P., Kasalkova N.S., Sajdl P., Kolska Z., Rimpelova S., Svorcik V. (2019). Antibacterial properties of angle-dependent nanopatterns on polystyrene. React. Funct. Polym..

[25] Orazi L., Pelaccia R., Siciliani V., Oubellaouch K. (2023). Ultrafast Laser Texturing to Improve Wettability of Polyimide (Kapton) Films. Precis. Eng..

[26] Lu X., Lu Q., Zhu Z., Yin J., Wang Z. (2007). Effect of Irradiation History on the Preparation of Laser Induced Periodic Microstructure on Polyimide Surface. Surf. Coatings Technol..

[27] Wang H., Deng D., Zhai Z., Yao Y. (2024). Laser-Processed Functional Surface Structures for Multi-Functional Applications—A Review. Precis. Eng..

[28] Alamri S., Fraggelakis F., Kunze T., Krupop B., Mincuzzi G., Kling R., Lasagni A. (2019). On the Interplay of DLIP and LIPSS Upon Ultra-Short Laser Pulse Irradiation. Materials.

[29] Bian J., Chen F., Ling H., Sun N., Hu J. (2022). Experimental and Modeling Study of Controllable Laser Lift-Off via Low-Fluence Multiscanning of Polyimide-Substrate Interface. Int. J. Heat Mass Transf..

[30] Sun X., Wang W., Mei X., Zhang C., Han F. (2025). High-Temperature Resistant Marking Patterns Prepared on Polyimide Film Using Femtosecond Laser. Opt. Laser Technol..

[31] Li M., Lu Q.H., Yin J., Qian Y., Wang Z.G. (2003). Effects of Post-Thermal Treatment on Preparation of Surface Microstructures Induced by Polarized Laser on Polyimide Film. Mater. Chem. Phys..

[32] Demiri V., Ehrhardt M., Lorenz P., Heinke R. (2023). Pulse Duration Dependent Laser-Induced Plasma Etching of Polyimide Using a High Repetition Rate Laser. Surfaces Interfaces.

[33] Ponnamma D., Sivakumar V., Popelka A., Hussein Y.H.A., Al-Maadeed S. (2019). Laser induced periodic surface structures on nano metal oxide filled polyvinylidene fluoride nanocomposites. Optik.

[34] Rebollar E., Perez S., Hernández M., Domingo C., Martin M., Ezquerra T.A., Garcia-Ruiz J.P., Castillejo M. (2014). Physicochemical modifications accompanying UV laser induced surface structures on poly(ethylene terephthalate) and their effect on adhesion of mesenchymal cells. Phys. Chem. Chem. Phys..

[35] Barb R.A., Hrelescu C., Dong L. (2014). Laser-induced periodic surface structures on polymers for formation of gold nanowires and activation of human cells. Appl. Phys. A.

[36] Yada S., Terakawa M. (2015). Femtosecond laser induced periodic surface structure on poly-L-lactic acid. Opt. Express.

[37] Rebollar E., Frischauf I., Olbrich M., Peterbauer T., Hering S., Preiner J., Hinterdorfer P., Romanin C., Heitz J. (2008). Proliferation of aligned mammalian cells on laser-nanostructured polystyrene. Biomaterials.

[38] Rebollar E., Sanz M., Perez S., Hernández M., Martín-Fabiani I., Rueda D.R., Ezquerra T.A., Domingo C., Castillejo M. (2012). Gold coatings on polymer laser induced periodic surface structures: Assessment as substrates for surface-enhanced Raman scattering. Phys. Chem. Chem. Phys..

[39] Collins B.A., Tumbleston J.R., Ade H. (2011). Miscibility, Crystallinity, and Phase Development in P3HT/PCBM Solar Cells: Toward an Enlightened Understanding of Device Morphology and Stability. J. Phys. Chem. Lett..

[40] Rodriguez-Rodriguez A., Rebollar E., Soccio M., Ezquerra T.A., Rueda D.R., Garcia-Ramos J.V., Castillejo M., Garcia-Gutierrez M.-C. (2015). Laser-Induced Periodic Surface Structures on Conjugated Polymers: Poly(3-hexylthiophene). Macromolecules.

[41] (2021). Geometrical Product Specifications (GPS)—Surface Texture: Profile—Part 2: Terms, Definitions and Surface Texture Parameters.

